# Supplementary material for: Single-cell transcriptome reveals the heterogeneity of malignant ductal cells and the prognostic value of REG4 and SPINK1 in primary pancreatic ductal adenocarcinoma
Source: PeerJ. 2024 May 28;12:e17350. doi: 10.7717/peerj.17350 (PMC11141562; doi:10.7717/peerj.17350)
Supplement: Supplemental Information 1 [file peerj-12-17350-s001.docx]

Supplementary Figures

Single-cell transcriptome reveals the heterogeneity of malignant ductal cells and the prognostic value of REG4 and SPINK1 in primary pancreatic ductal adenocarcinoma

Yutian Ji, Qianhui Xu, Weilin Wang^*^

**Supplementary Figure 1 and 2.** Characterization of single-cell RNA sequencing. Initial quality control of scRNA-seq for cells from each sample. Cells with poor quality were filtered and analyzed the positive associations between detected gene counts and sequencing depth.
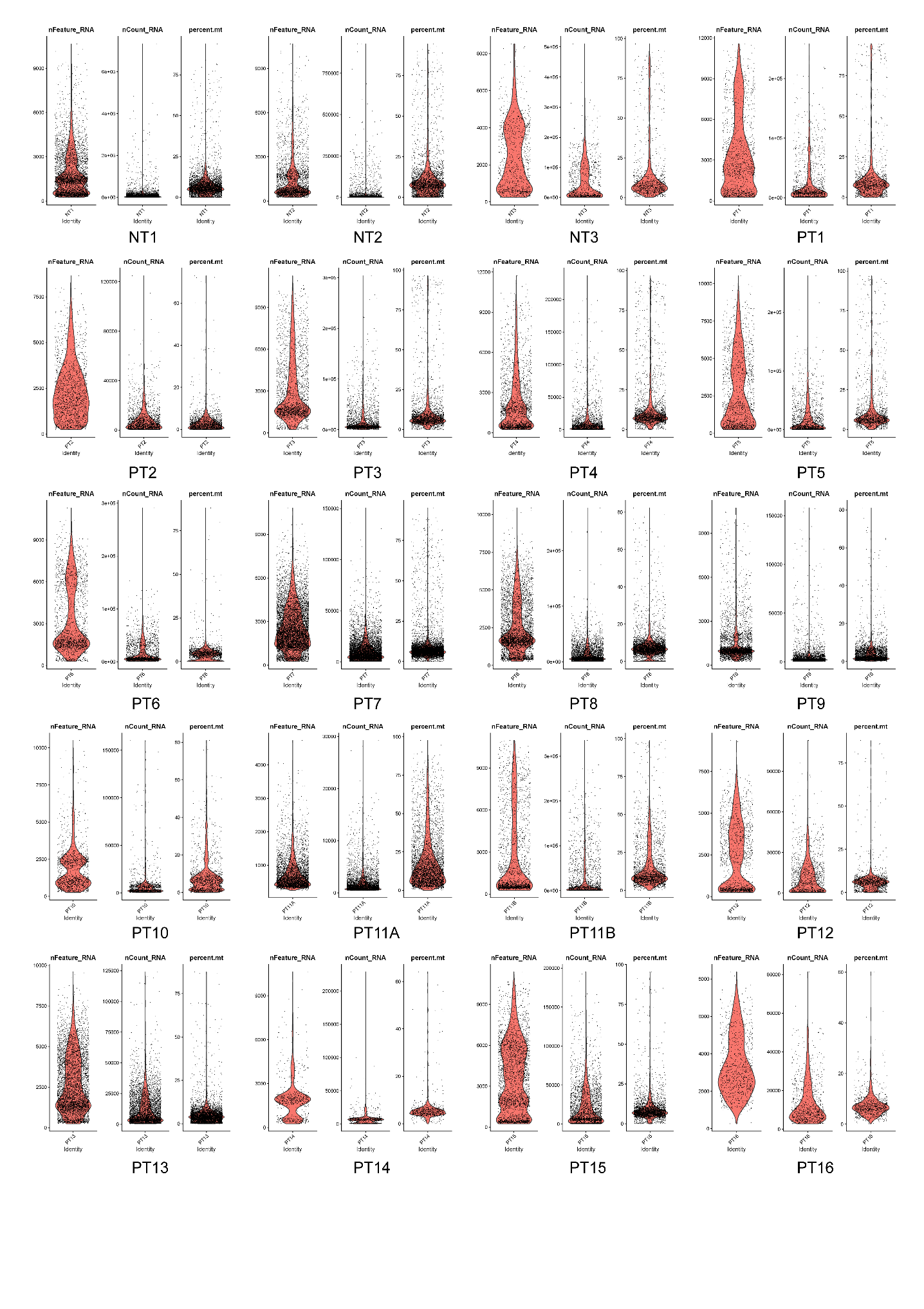


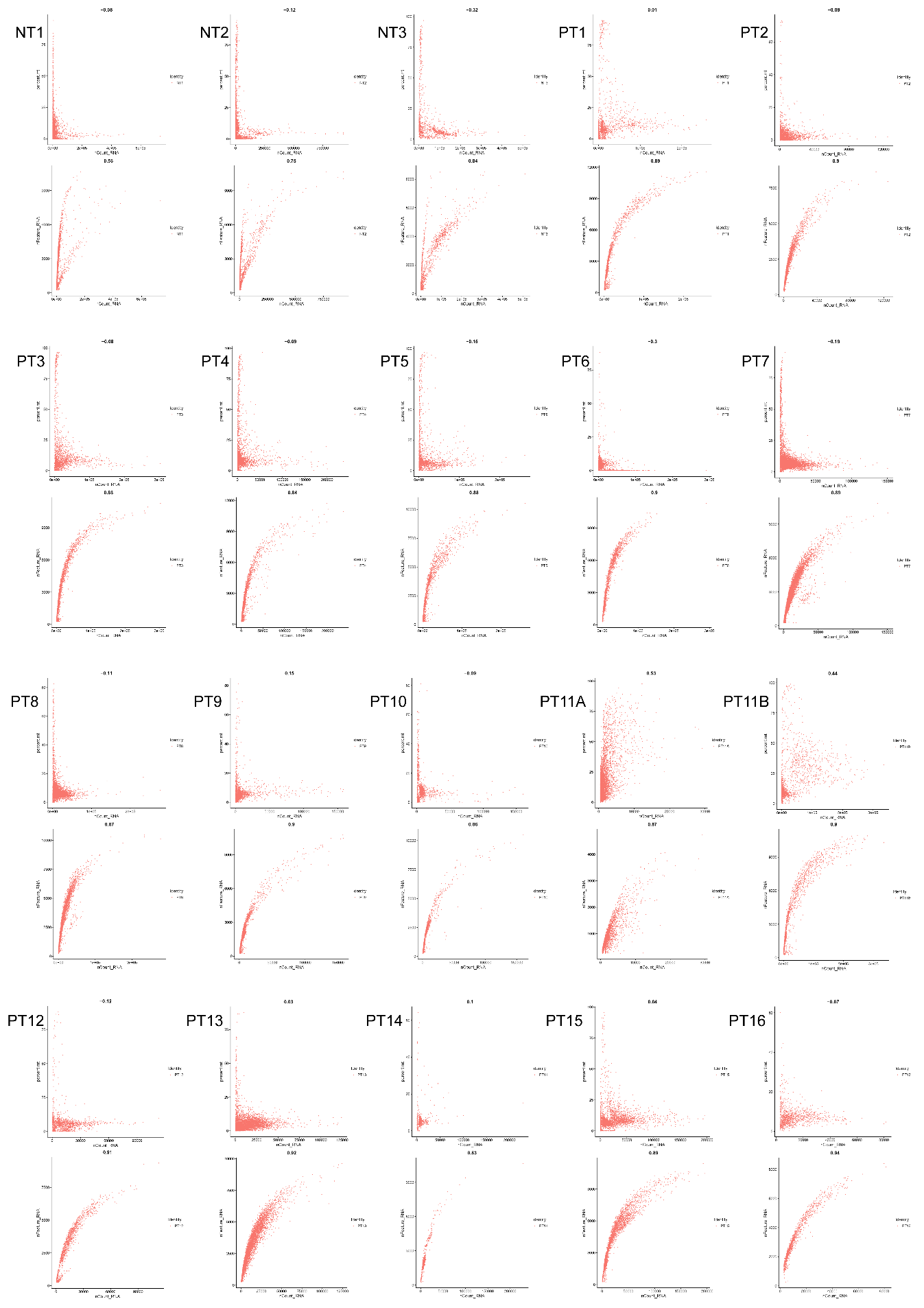


**Supplementary Figure 3.** Screening of marker genes and investigation of cellular components. (a) Gene markers with significant differences were identified across cells and drawn the characteristic variance diagram for each PDAC lesion; (b) PCA and ClusterTree.
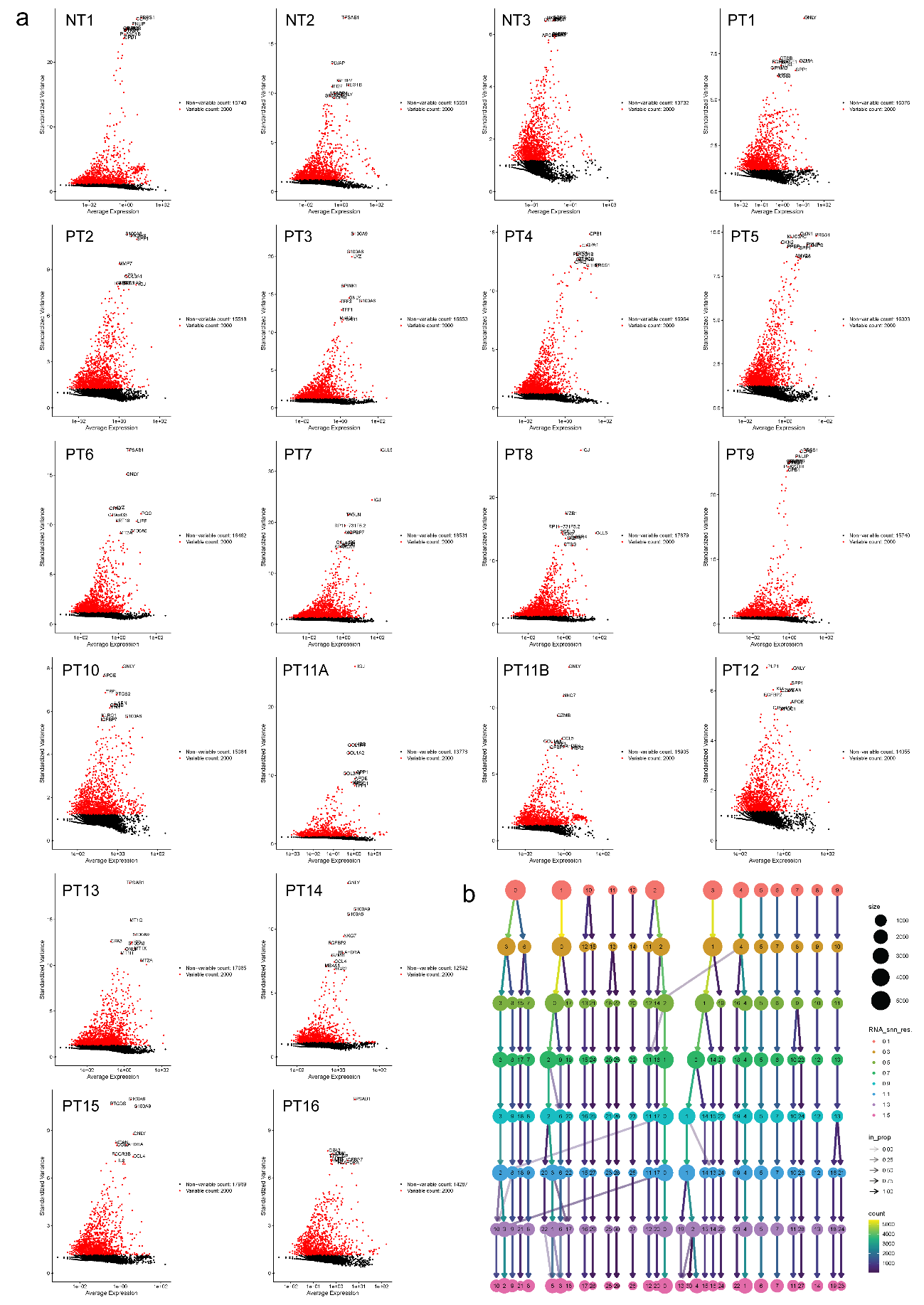


**Supplementary Figure 4.** Intratumoral heterogeneity of PDCA cells in different lesions. Additional filtering methods like (a) DoubletFinder and (b) cell cycle phase distribution analysis was applied before t-SNE analysis; (c)26 main subclusters were identified with t-SNE analysis; (d) t-SNE plot showed the 26 subclusters from 16 PDAC samples and 3 normal tissues.
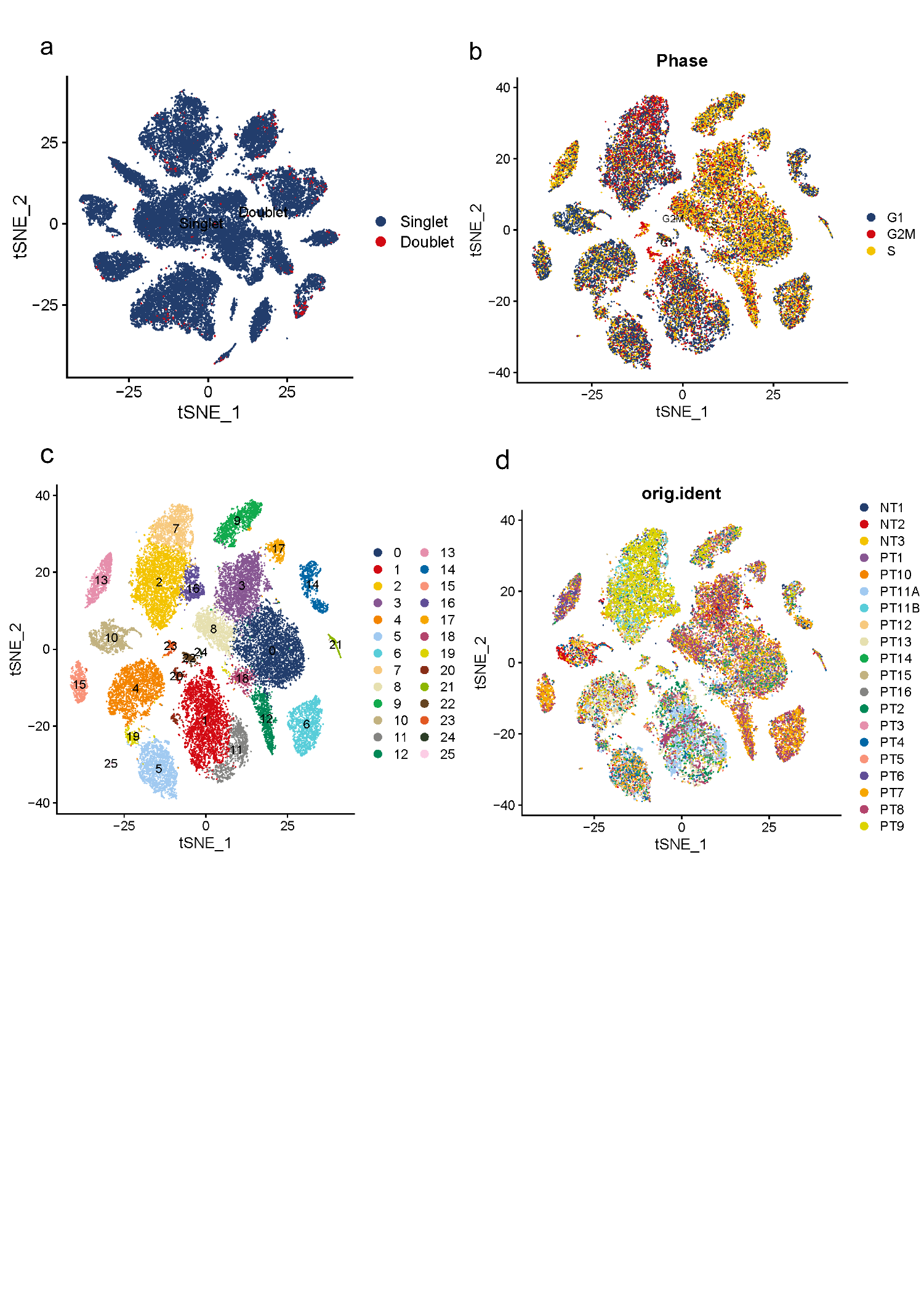


**Supplementary Figure 5.** (a) Distribution of 5 malignant ductal cell types in each PDAC sample; (b) Enrichment of KEGG pathway terms in each subgroup.


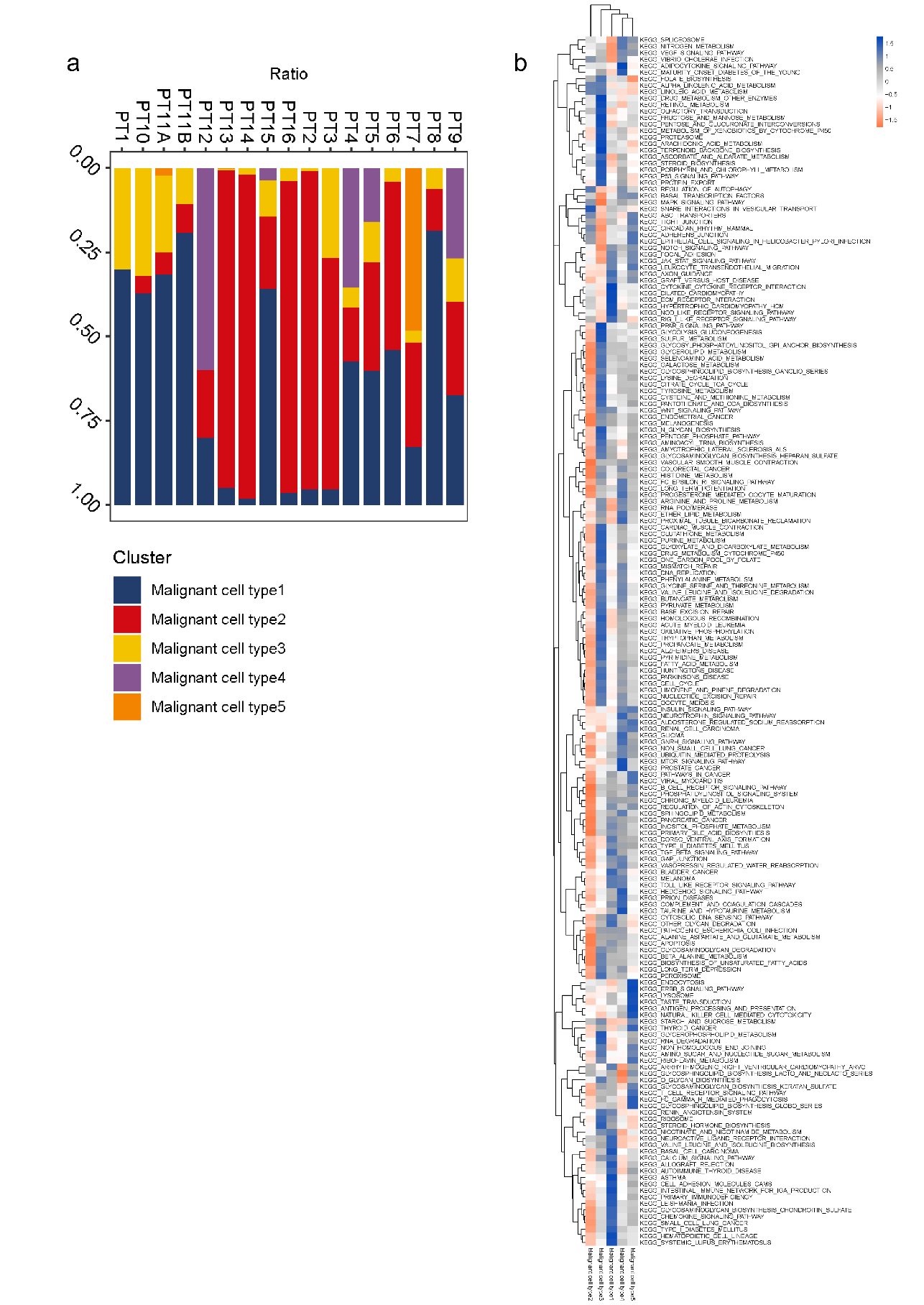


**Supplementary Figure 6.** Pseudotime trajectory analysis of malignant ductal cells in each PDAC sample. (a) pseudotime trajectory for 16 PDAC samples; (b) pseudotime trajectory for each PDAC sample.


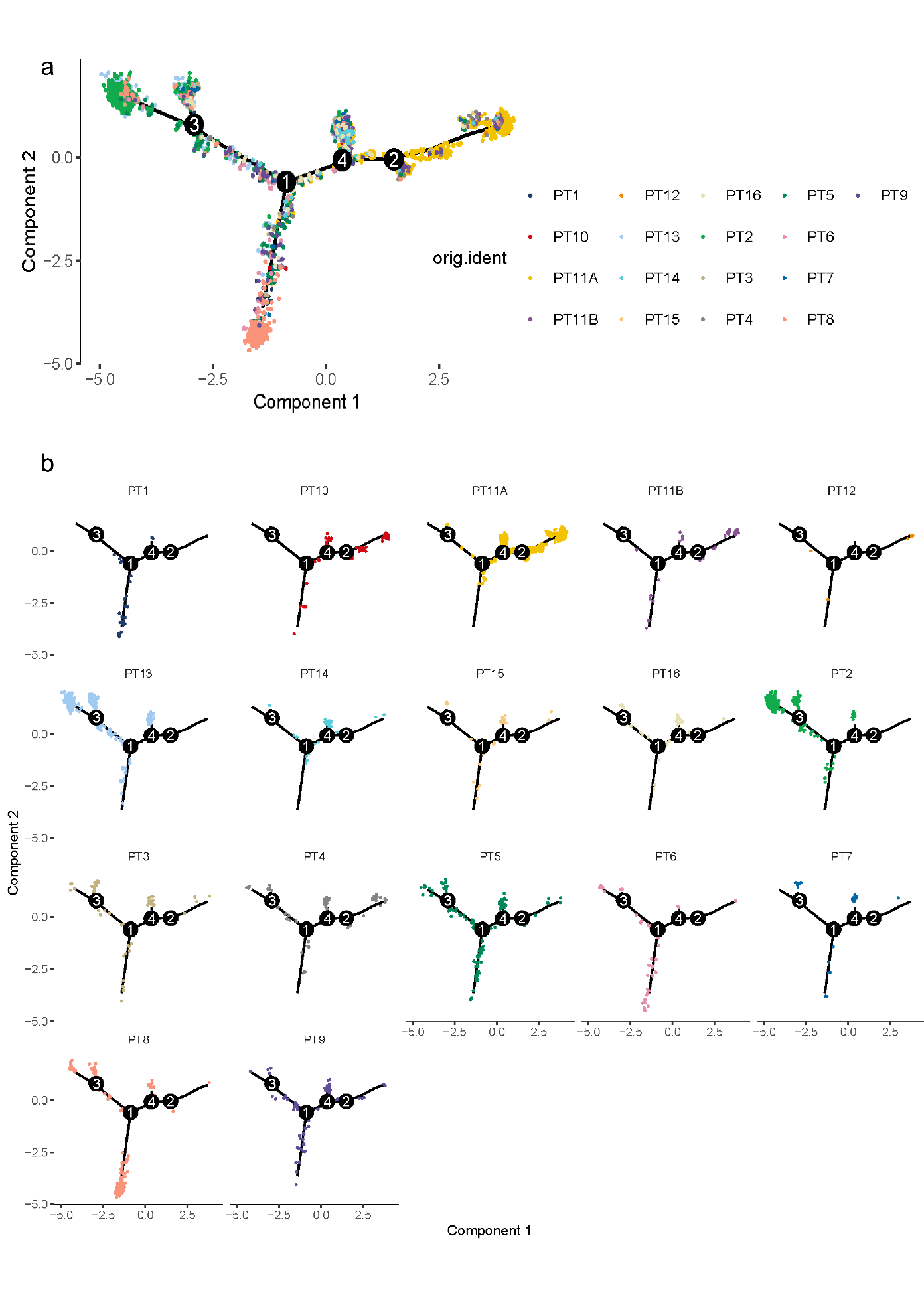


**Supplementary Figure 7.** Distinct subtypes of T cells and myeloid cells in PDAC lesions. (a) t-SNE plots showing three subtypes for T cells; (b, c) Violin plots and feature plots for marker genes for T cell subtypes; (d) t-SNE plots showing three subtypes for myeloid cells; (e, f) Violin plots and feature plots for marker genes for myeloid cell subtypes.
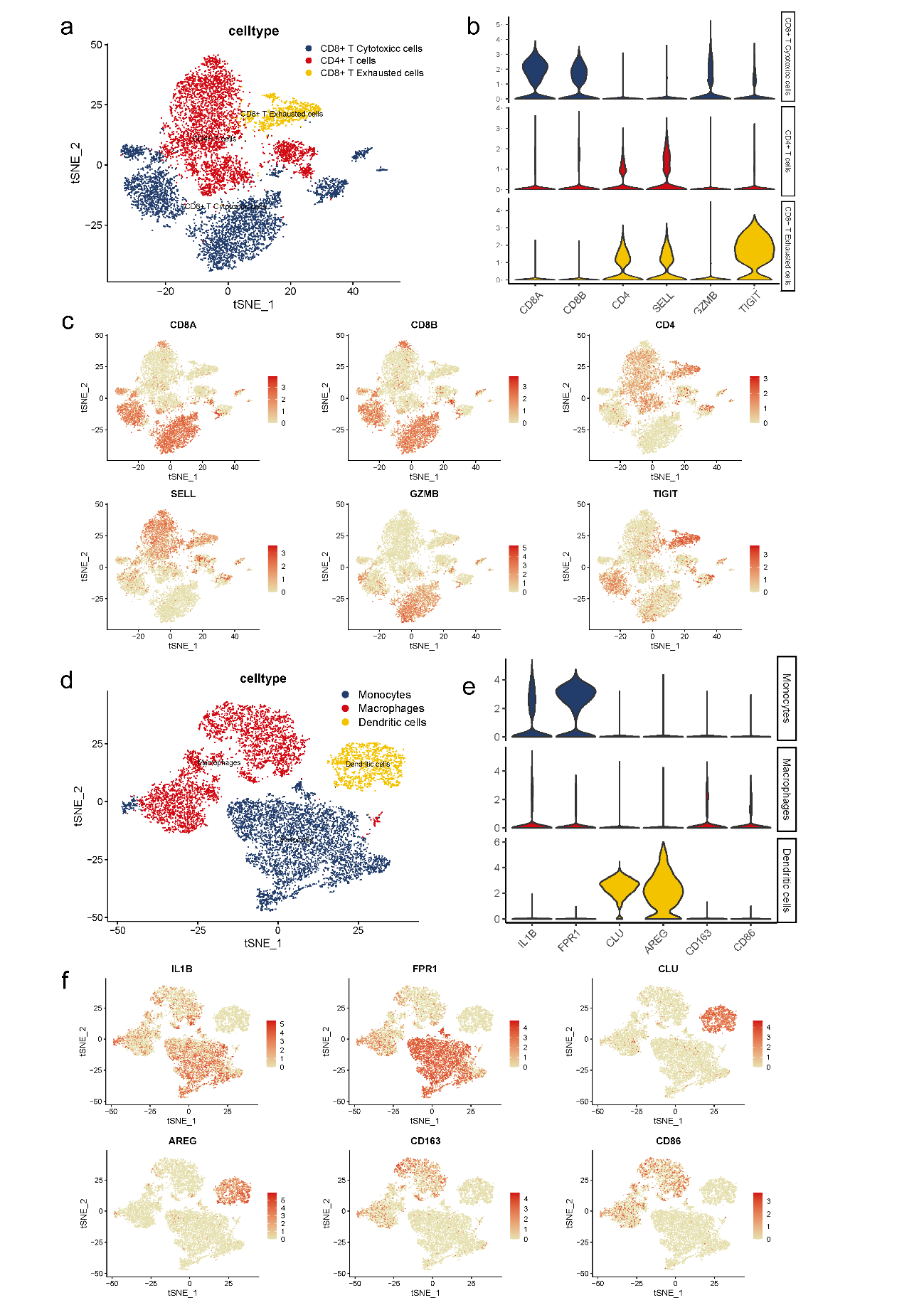


**Supplementary Figure 8.** Heatmap plots of cell-cell interactions between malignant ductal cells and stromal cells. (a) Under count value; (b) Under log-count value.
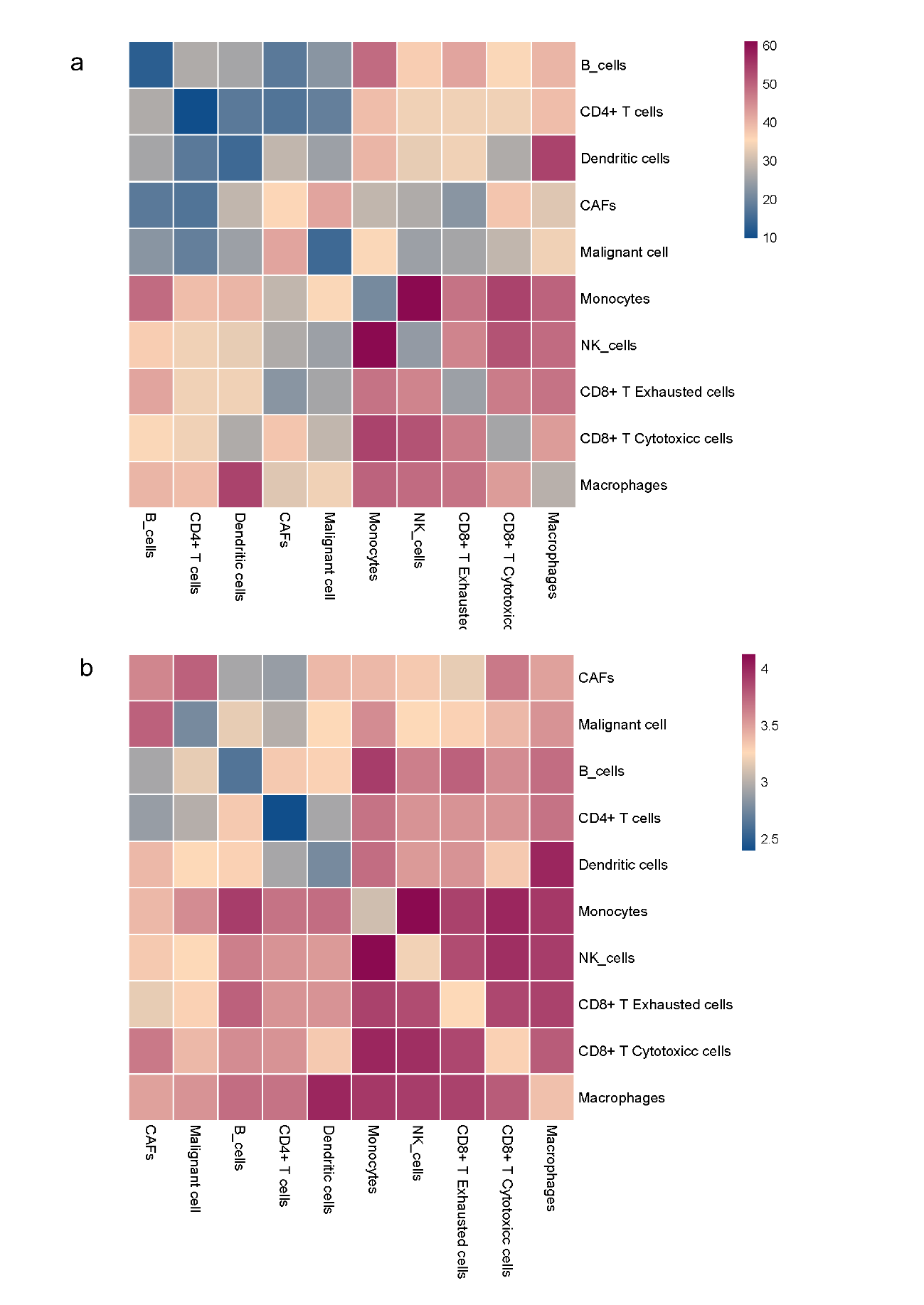


**Supplementary Figure 9.** Expression patterns of selected potential DEGs. Differentially expressed proteins of REG4 in normal (a) and PDAC samples (b); Differentially expressed proteins of SPINK1 in normal (c) and PDAC samples (d) in HPA database.
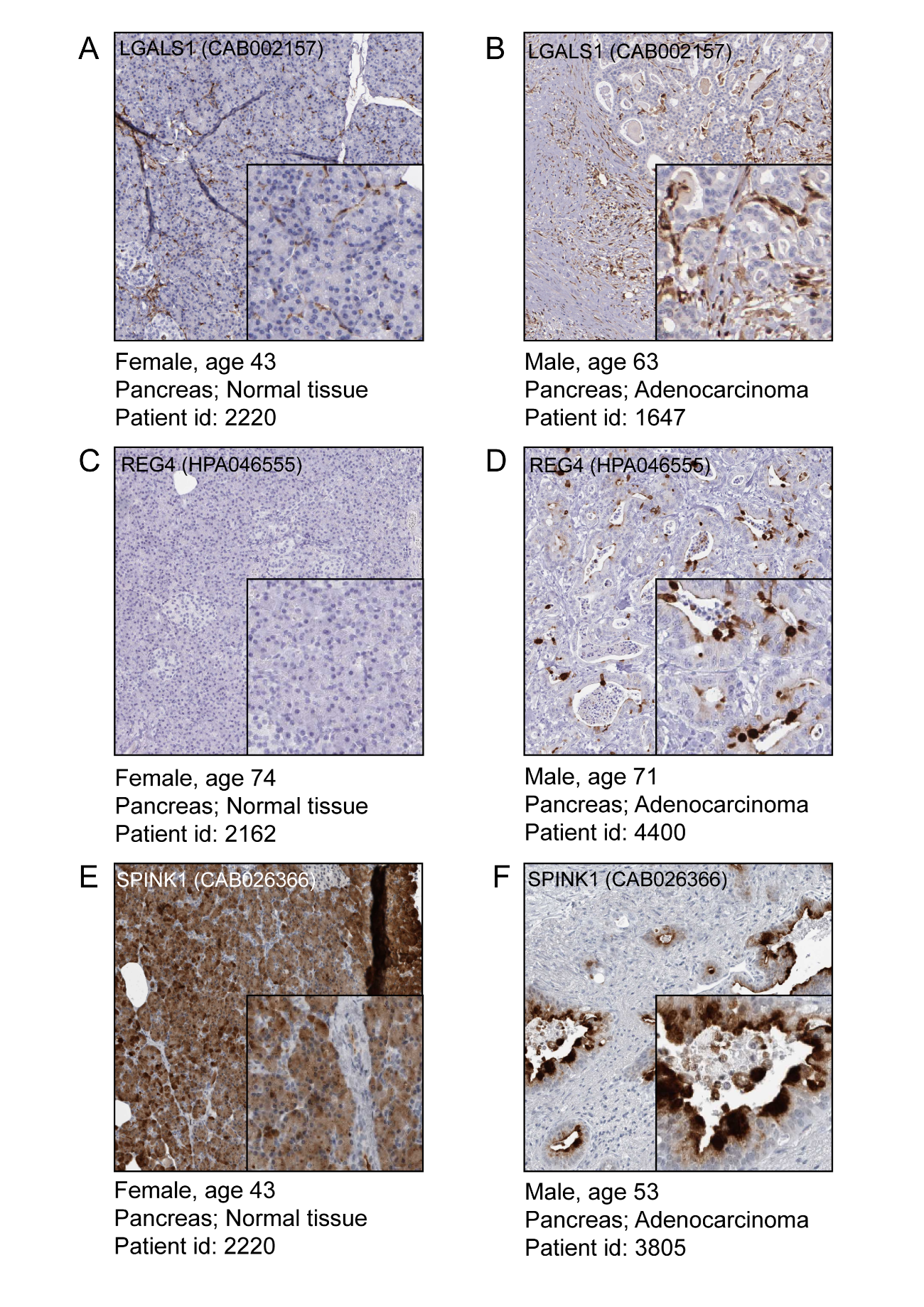


a

b

c

d

**Supplementary Figure 10.** Correlation between gene expression and immune infiltration in PDAC. (a, b) Correlation between levels of various immune cell types (B cell, CD8+ T cell, CD4+ T cell, macrophage, neutrophil and dendritic cell) and gene expression of REG4 and SPINK1, respectively; (c) Correlation between well-defined immune inhibiting molecules and gene expression of SPINK1.

a

b

c


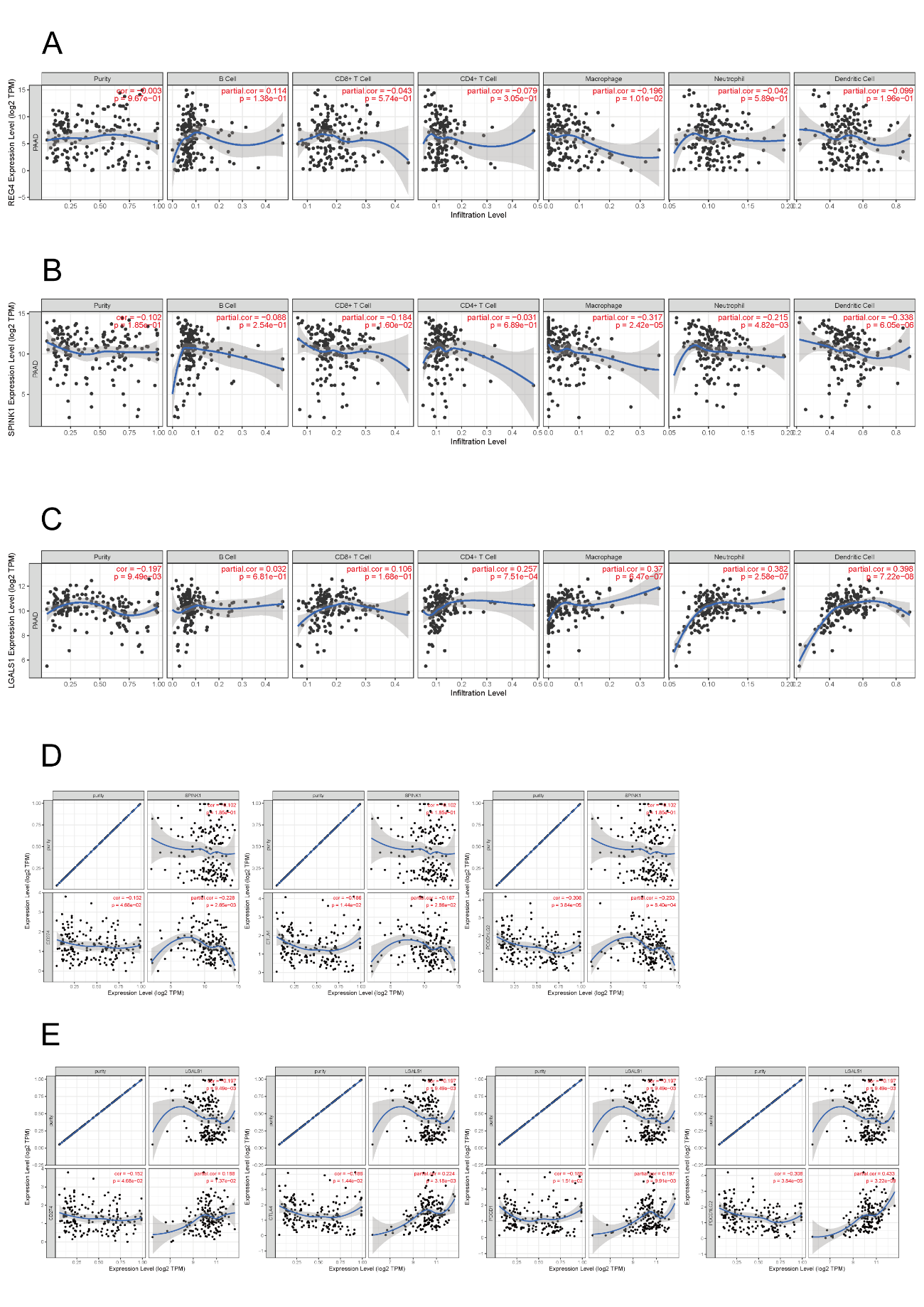

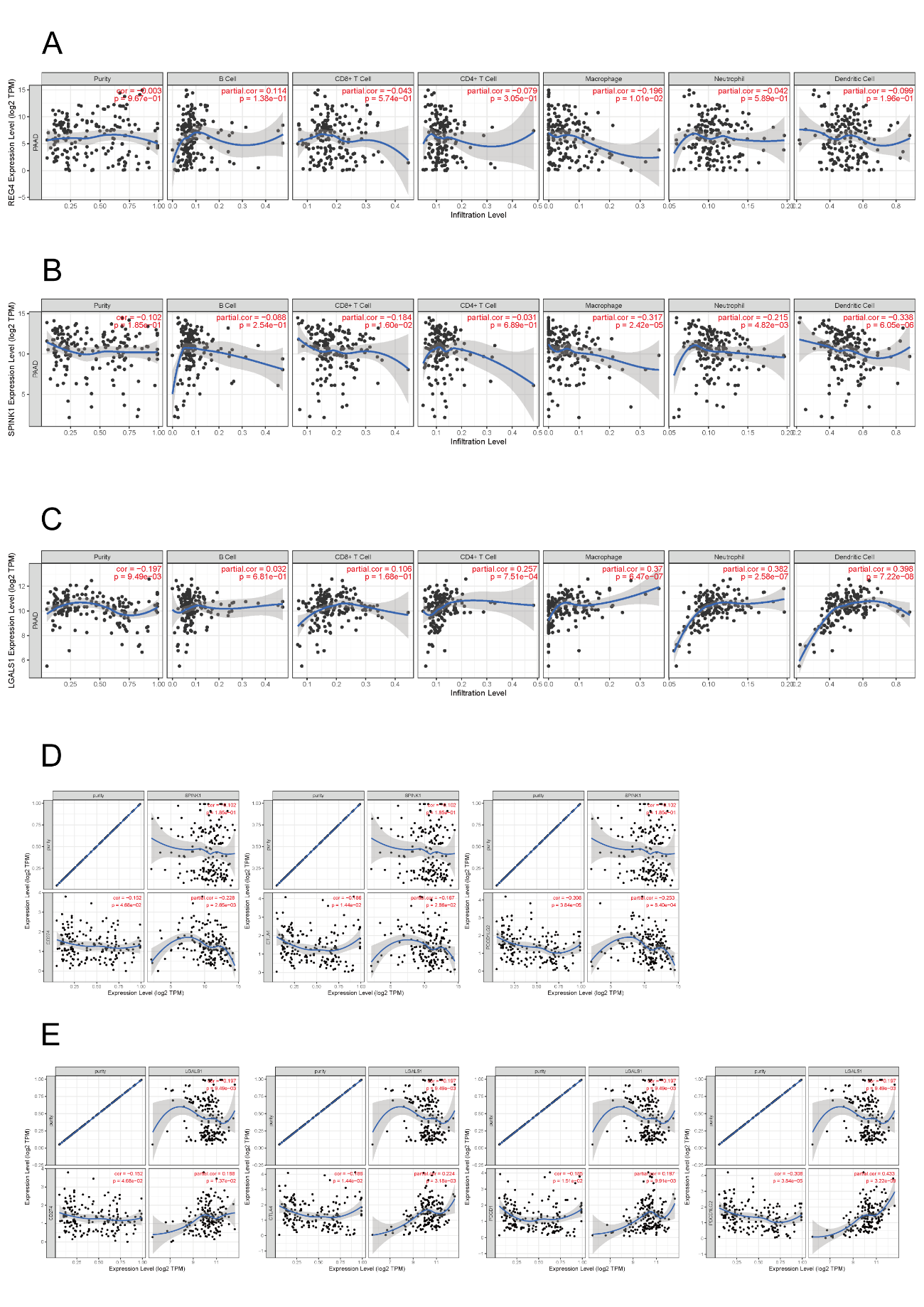


**Supplementary Figure 11.** Correlation between gene expression and overall survival of PDAC patients. (a, b) Kaplan-Meier survival analysis revealed correlation between levels of overall survival of PDAC patients and gene expression of REG4 and SPINK1, respectively.


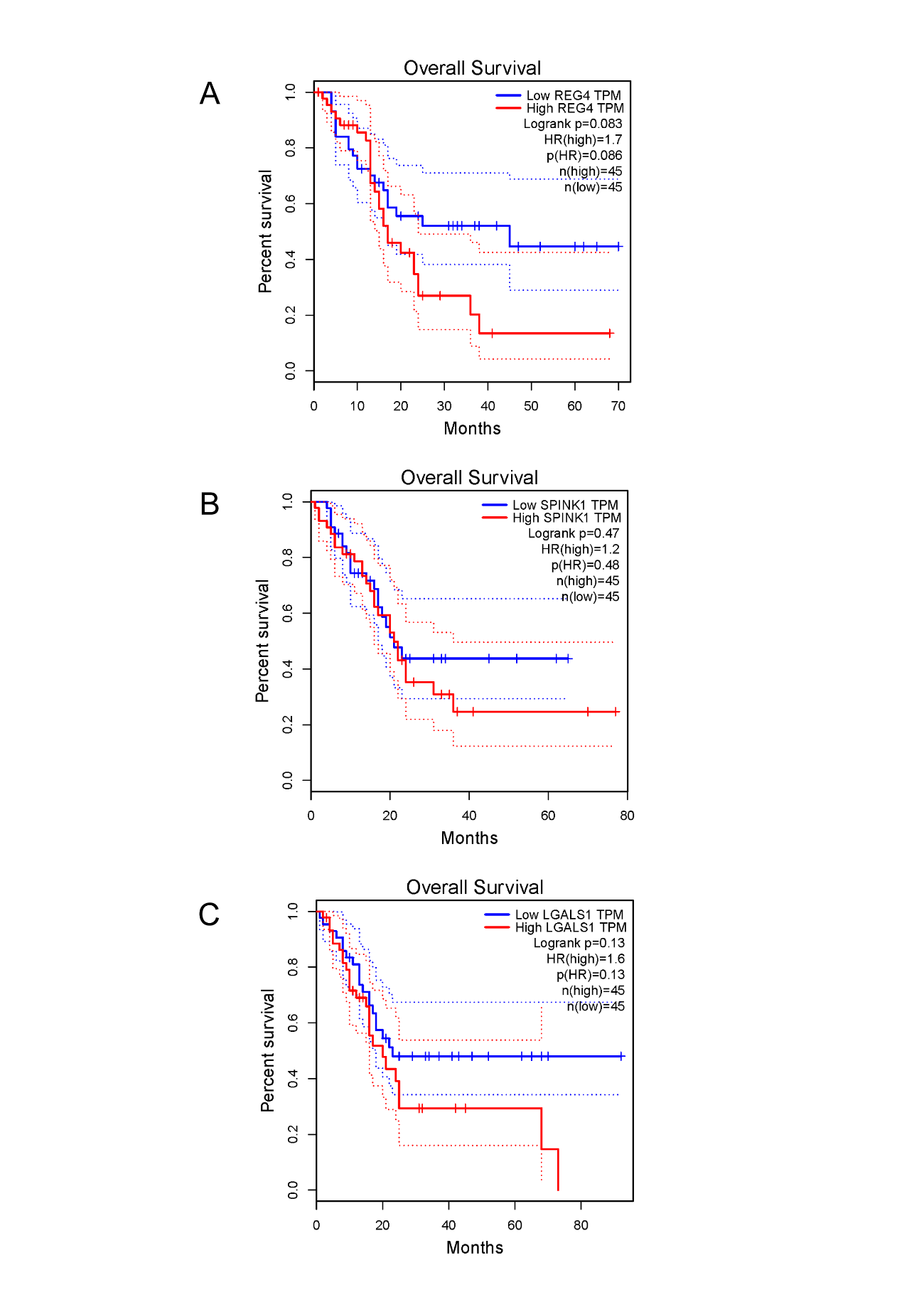


a

b
