# Supplementary material for: Single-cell transcriptome reveals the heterogeneity of malignant ductal cells and the prognostic value of REG4 and SPINK1 in primary pancreatic ductal adenocarcinoma
Source: PeerJ. 2024 May 28;12:e17350. doi: 10.7717/peerj.17350 (PMC11141562; doi:10.7717/peerj.17350)

Cropped blots of REG4 from Figure 6

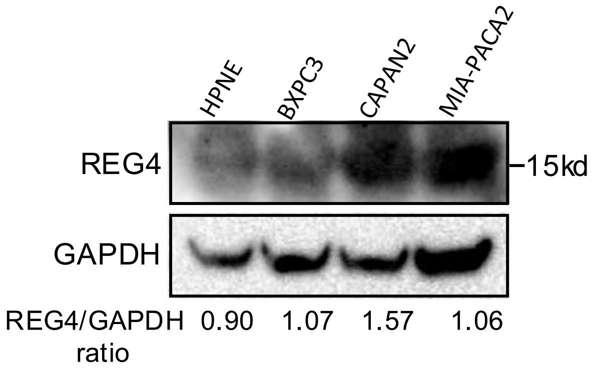

Full length blots

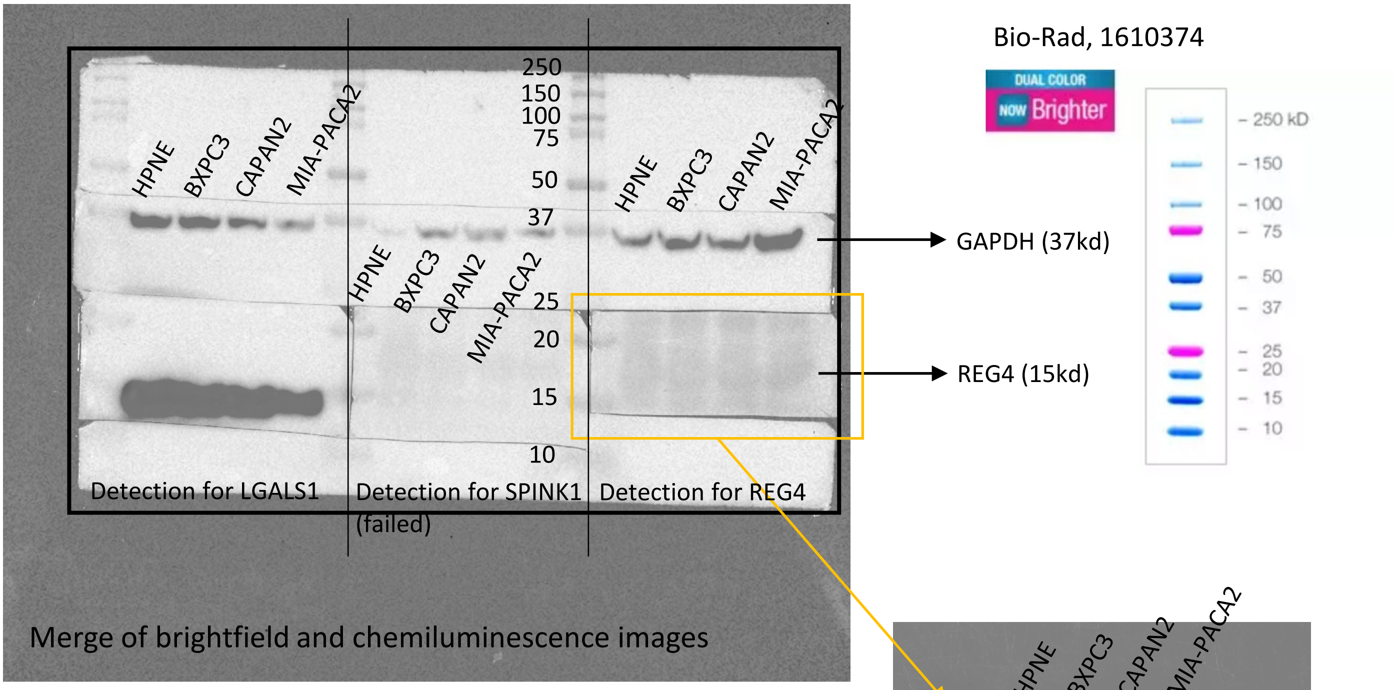

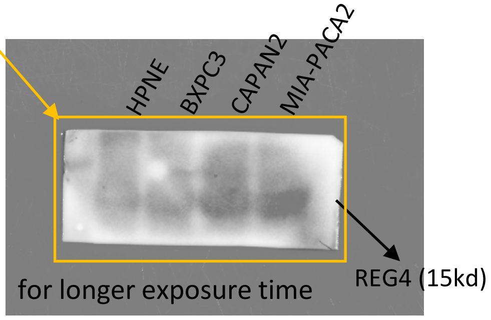

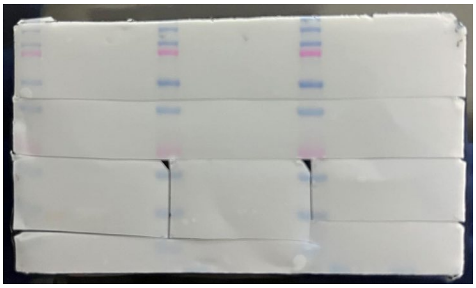

Cropped blots of SPINK1 from Figure 6

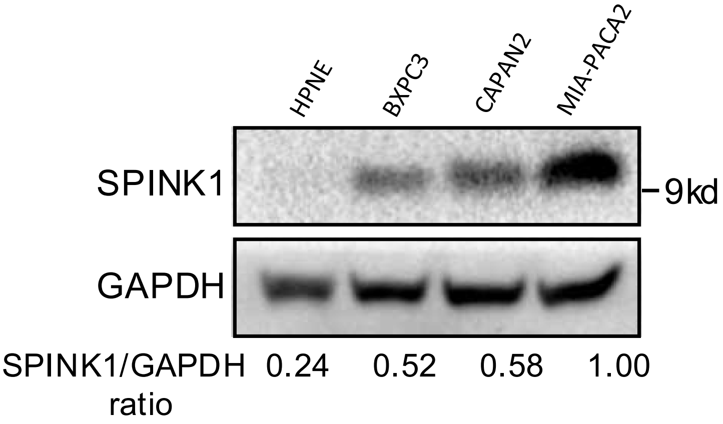

Full length blots

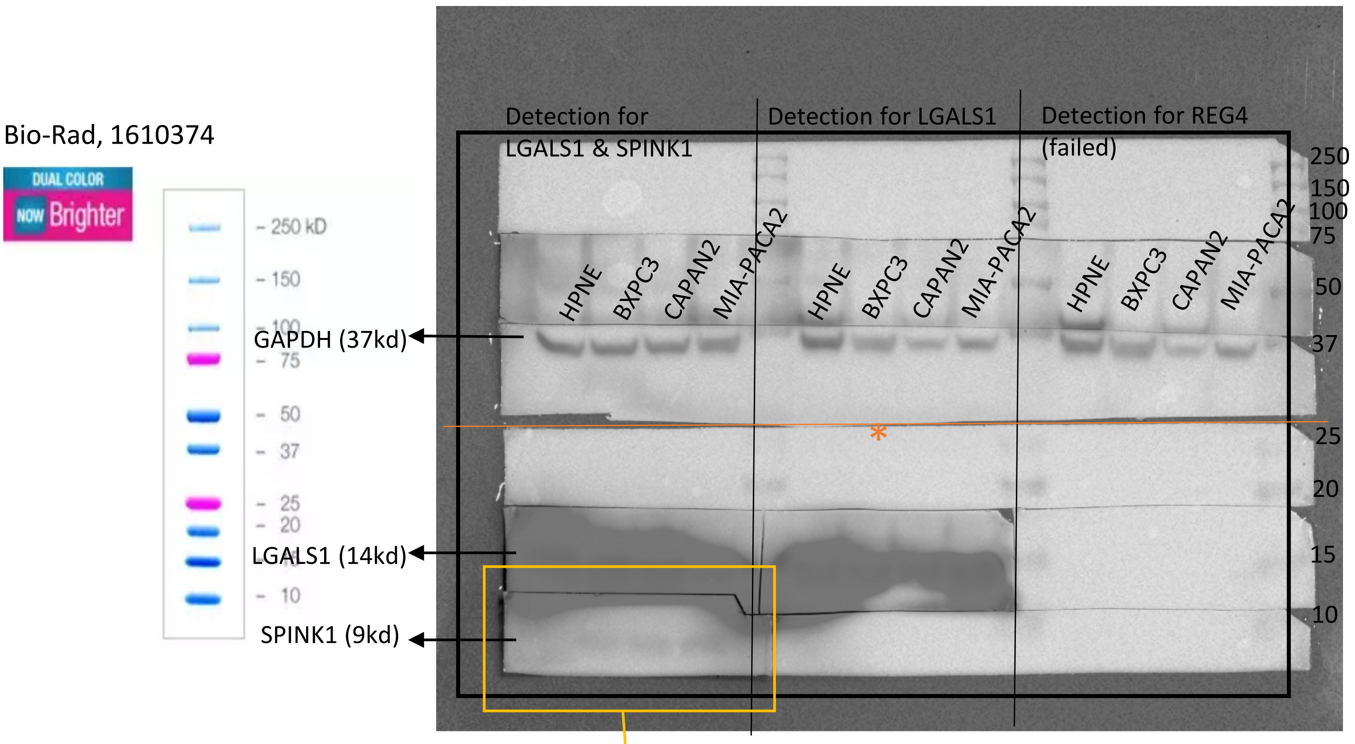

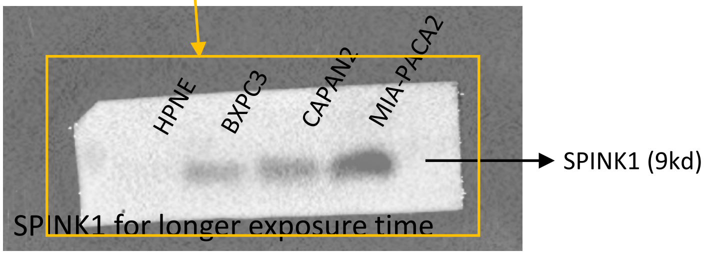

*The PVDF membrane is not continuous here, because the upper part and the lower part were transferred separately for different time.

Cropped blots of LGALS1 from Figure 6

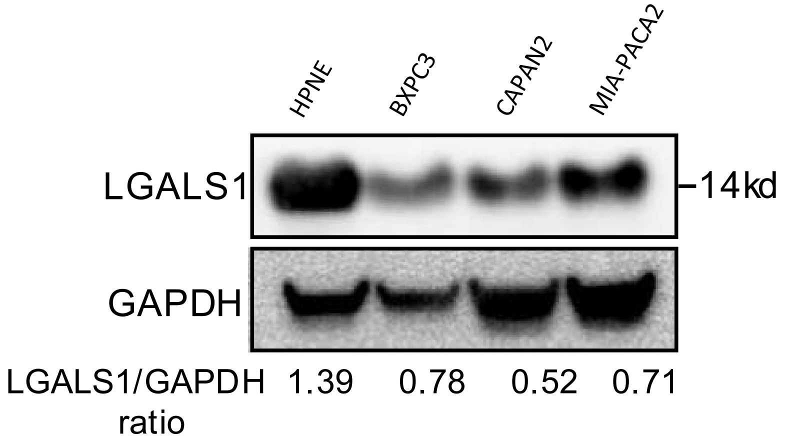

Full length blots

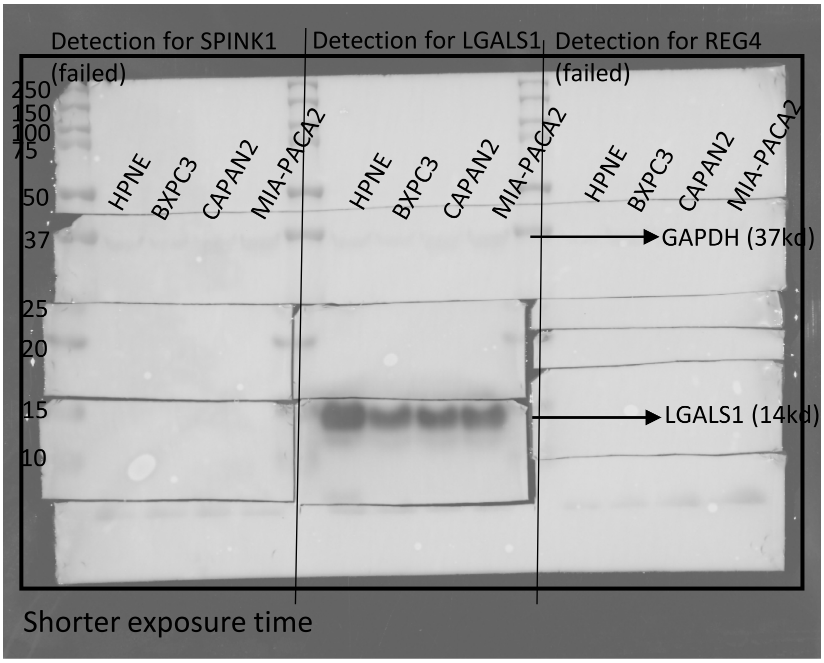

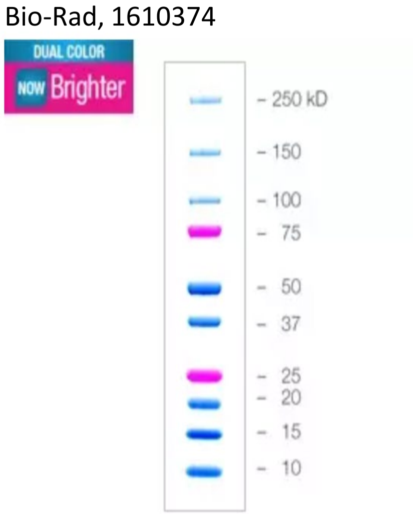

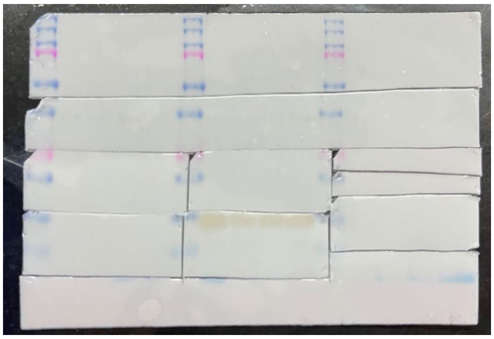

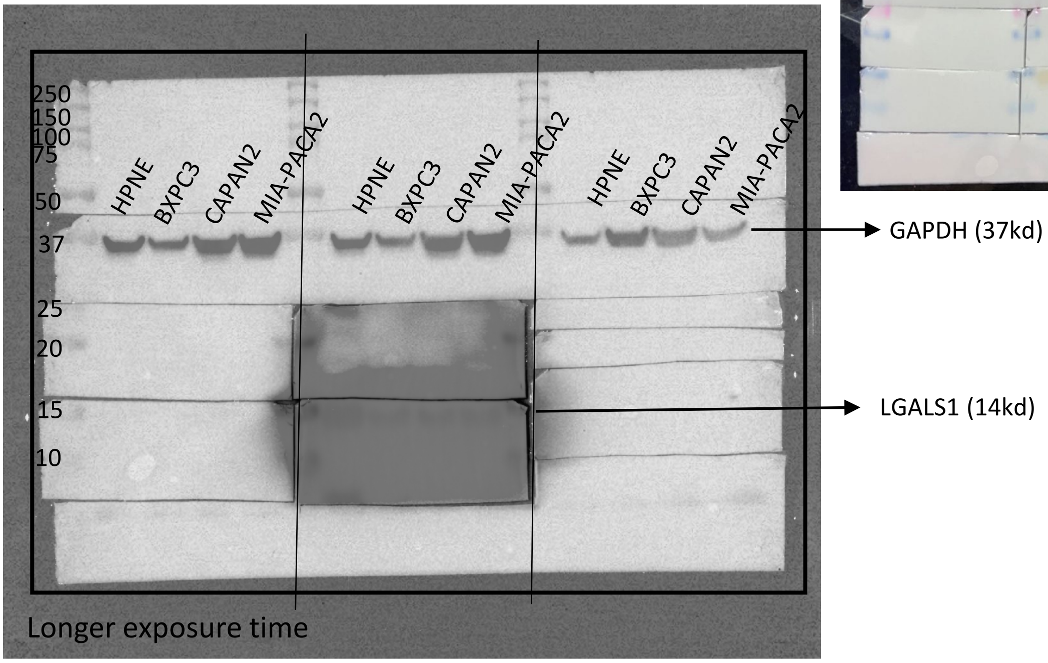

Supplement: Supplemental Information 3 — Cropped original blots in Figs. 6A–6C are shown in folders named REG4, SPINK1, LGALS1, respectively. Corresponding uncropped blots are shown in a PDF file named “uncropped blots”, where molecular weight ladders and target protein are clearly presented. Since small molecular weights of our target proteins (especially for SPINK1 ∼9kd), we did not get enough satisfying replicates in WB quantitative analysis. So relative mRNA levels were used to quantify expression of each gene in cell lines in Figs. 6A–6C (upper part), with WB results as a supplement reference (lower part). So only one WB result for each protein is provided. [file peerj-12-17350-s003.zip › uncropped blots.pdf]
