# Supplementary figures and images for: Single-cell transcriptome reveals the heterogeneity of malignant ductal cells and the prognostic value of REG4 and SPINK1 in primary pancreatic ductal adenocarcinoma
Source: PeerJ. 2024 May 28;12:e17350. doi: 10.7717/peerj.17350 (PMC11141562; doi:10.7717/peerj.17350)

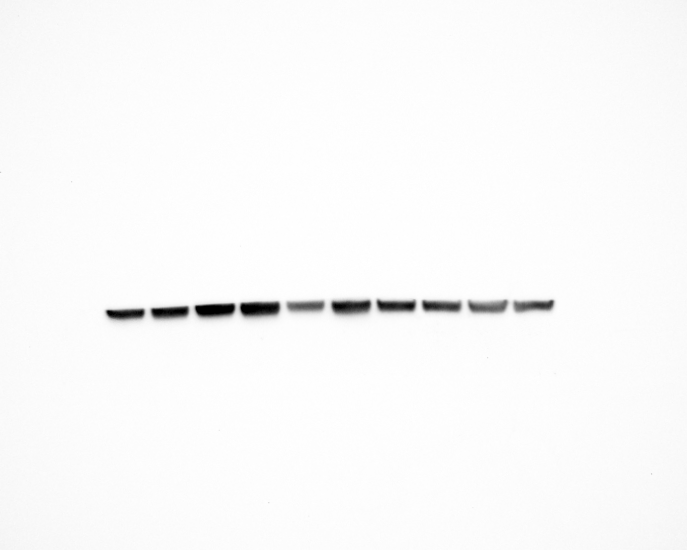

Supplement: Supplemental Information 3 — Cropped original blots in Figs. 6A–6C are shown in folders named REG4, SPINK1, LGALS1, respectively. Corresponding uncropped blots are shown in a PDF file named “uncropped blots”, where molecular weight ladders and target protein are clearly presented. Since small molecular weights of our target proteins (especially for SPINK1 ∼9kd), we did not get enough satisfying replicates in WB quantitative analysis. So relative mRNA levels were used to quantify expression of each gene in cell lines in Figs. 6A–6C (upper part), with WB results as a supplement reference (lower part). So only one WB result for each protein is provided. [file peerj-12-17350-s003.zip › LGALS1/jyt 2022-08-15 17h41m06s(Chemiluminescence).raw16.tif]

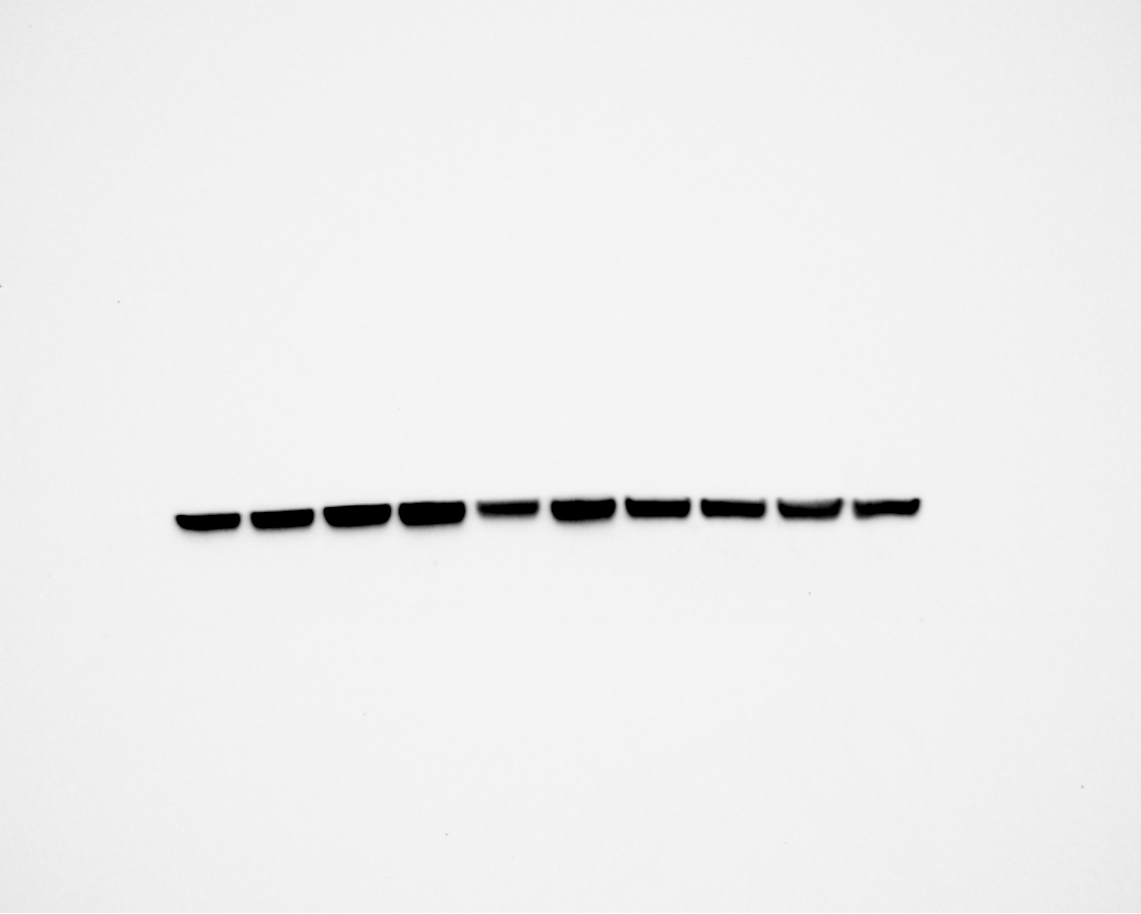

Supplement: Supplemental Information 3 — Cropped original blots in Figs. 6A–6C are shown in folders named REG4, SPINK1, LGALS1, respectively. Corresponding uncropped blots are shown in a PDF file named “uncropped blots”, where molecular weight ladders and target protein are clearly presented. Since small molecular weights of our target proteins (especially for SPINK1 ∼9kd), we did not get enough satisfying replicates in WB quantitative analysis. So relative mRNA levels were used to quantify expression of each gene in cell lines in Figs. 6A–6C (upper part), with WB results as a supplement reference (lower part). So only one WB result for each protein is provided. [file peerj-12-17350-s003.zip › LGALS1/jyt 2022-08-15 17h41m06s(Chemiluminescence).tif]

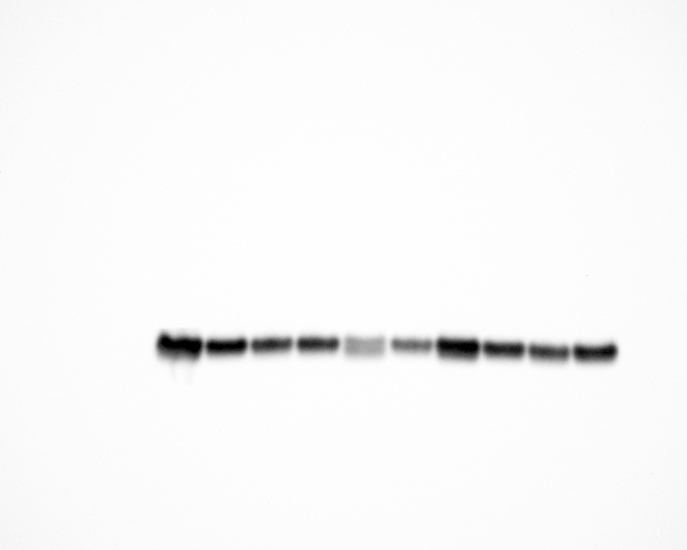

Supplement: Supplemental Information 3 — Cropped original blots in Figs. 6A–6C are shown in folders named REG4, SPINK1, LGALS1, respectively. Corresponding uncropped blots are shown in a PDF file named “uncropped blots”, where molecular weight ladders and target protein are clearly presented. Since small molecular weights of our target proteins (especially for SPINK1 ∼9kd), we did not get enough satisfying replicates in WB quantitative analysis. So relative mRNA levels were used to quantify expression of each gene in cell lines in Figs. 6A–6C (upper part), with WB results as a supplement reference (lower part). So only one WB result for each protein is provided. [file peerj-12-17350-s003.zip › LGALS1/jyt 2022-08-15 17h42m34s lgals1(Chemiluminescence).raw16.tif]

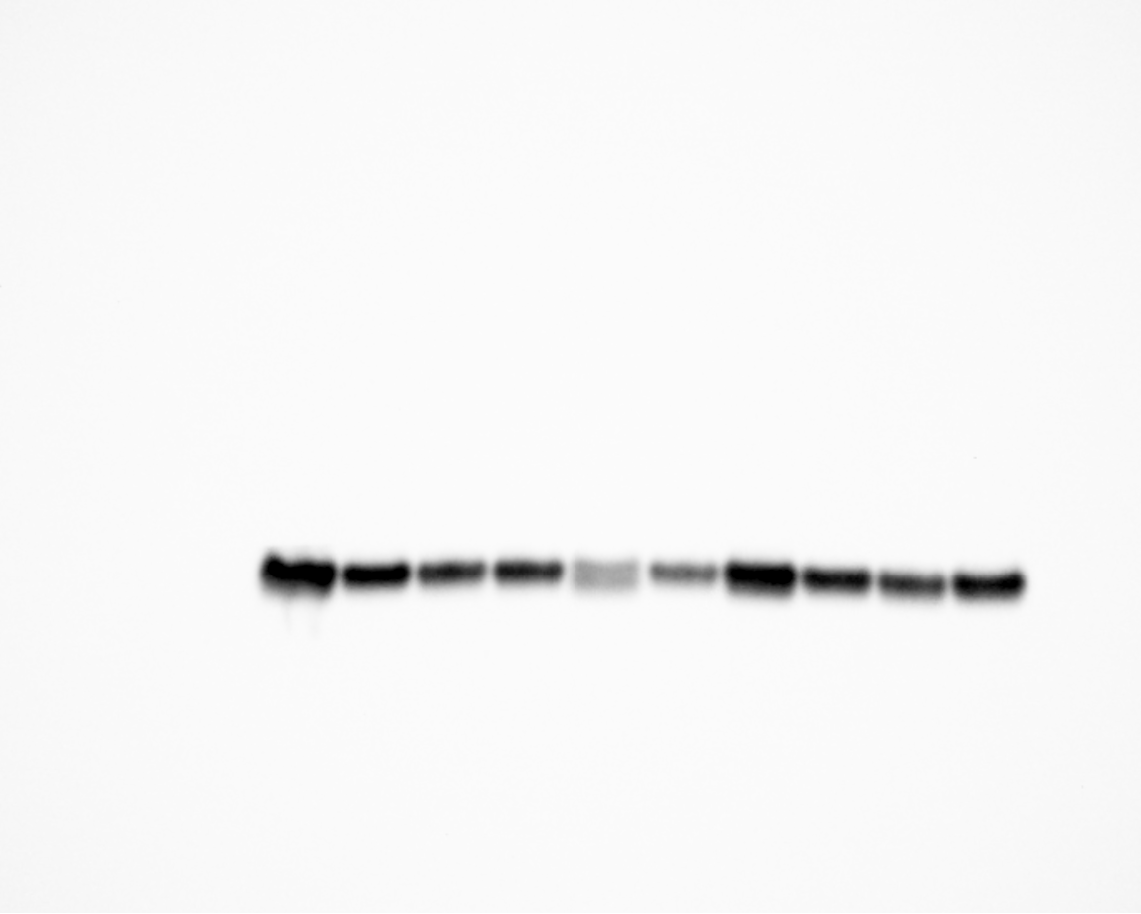

Supplement: Supplemental Information 3 — Cropped original blots in Figs. 6A–6C are shown in folders named REG4, SPINK1, LGALS1, respectively. Corresponding uncropped blots are shown in a PDF file named “uncropped blots”, where molecular weight ladders and target protein are clearly presented. Since small molecular weights of our target proteins (especially for SPINK1 ∼9kd), we did not get enough satisfying replicates in WB quantitative analysis. So relative mRNA levels were used to quantify expression of each gene in cell lines in Figs. 6A–6C (upper part), with WB results as a supplement reference (lower part). So only one WB result for each protein is provided. [file peerj-12-17350-s003.zip › LGALS1/jyt 2022-08-15 17h42m34s lgals1(Chemiluminescence).tif]

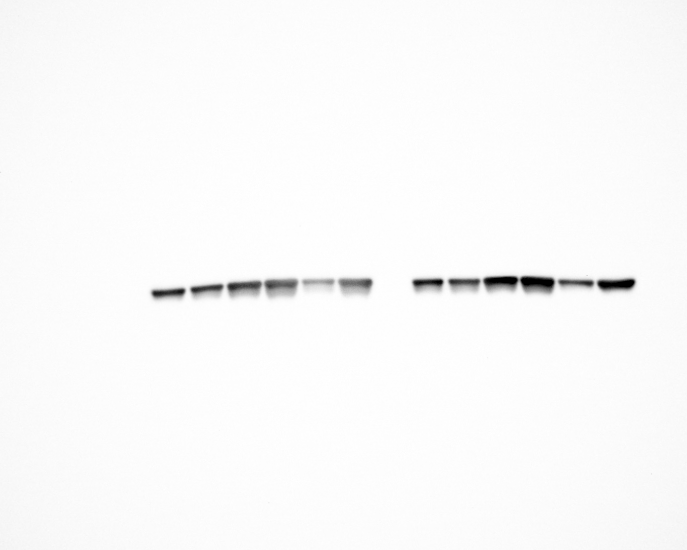

Supplement: Supplemental Information 3 — Cropped original blots in Figs. 6A–6C are shown in folders named REG4, SPINK1, LGALS1, respectively. Corresponding uncropped blots are shown in a PDF file named “uncropped blots”, where molecular weight ladders and target protein are clearly presented. Since small molecular weights of our target proteins (especially for SPINK1 ∼9kd), we did not get enough satisfying replicates in WB quantitative analysis. So relative mRNA levels were used to quantify expression of each gene in cell lines in Figs. 6A–6C (upper part), with WB results as a supplement reference (lower part). So only one WB result for each protein is provided. [file peerj-12-17350-s003.zip › REG4/jyt 2022-08-12 17h15m03s gapdh panc cell(Chemiluminescence).raw16.tif]

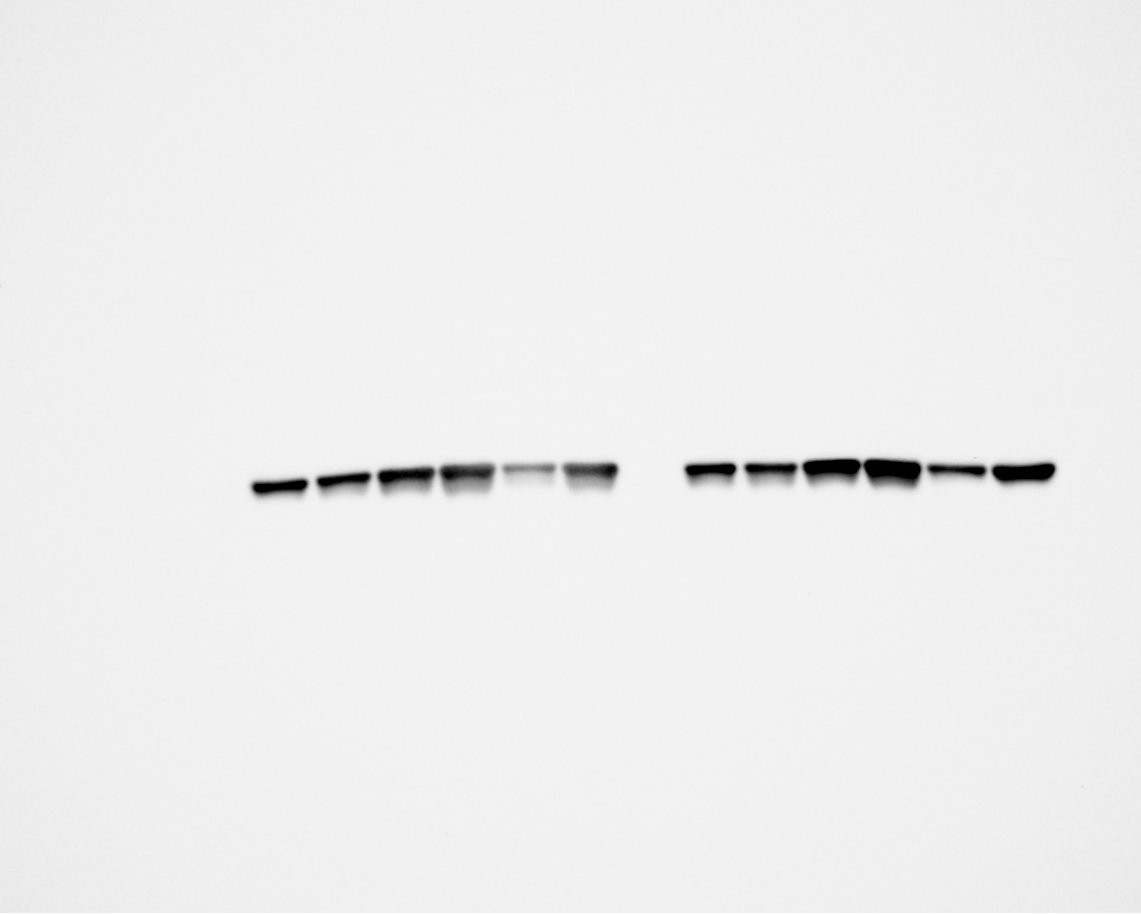

Supplement: Supplemental Information 3 — Cropped original blots in Figs. 6A–6C are shown in folders named REG4, SPINK1, LGALS1, respectively. Corresponding uncropped blots are shown in a PDF file named “uncropped blots”, where molecular weight ladders and target protein are clearly presented. Since small molecular weights of our target proteins (especially for SPINK1 ∼9kd), we did not get enough satisfying replicates in WB quantitative analysis. So relative mRNA levels were used to quantify expression of each gene in cell lines in Figs. 6A–6C (upper part), with WB results as a supplement reference (lower part). So only one WB result for each protein is provided. [file peerj-12-17350-s003.zip › REG4/jyt 2022-08-12 17h15m03s gapdh panc cell(Chemiluminescence).tif]

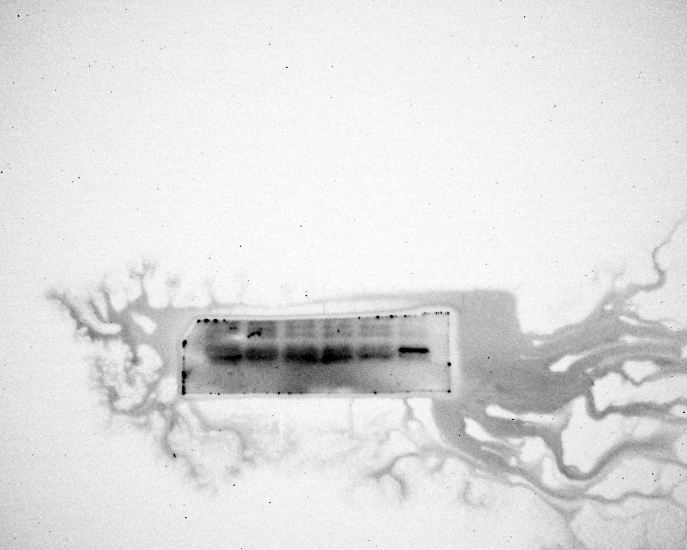

Supplement: Supplemental Information 3 — Cropped original blots in Figs. 6A–6C are shown in folders named REG4, SPINK1, LGALS1, respectively. Corresponding uncropped blots are shown in a PDF file named “uncropped blots”, where molecular weight ladders and target protein are clearly presented. Since small molecular weights of our target proteins (especially for SPINK1 ∼9kd), we did not get enough satisfying replicates in WB quantitative analysis. So relative mRNA levels were used to quantify expression of each gene in cell lines in Figs. 6A–6C (upper part), with WB results as a supplement reference (lower part). So only one WB result for each protein is provided. [file peerj-12-17350-s003.zip › REG4/jyt 2022-08-12 17h23m02s reg4(Chemiluminescence).raw16.tif]

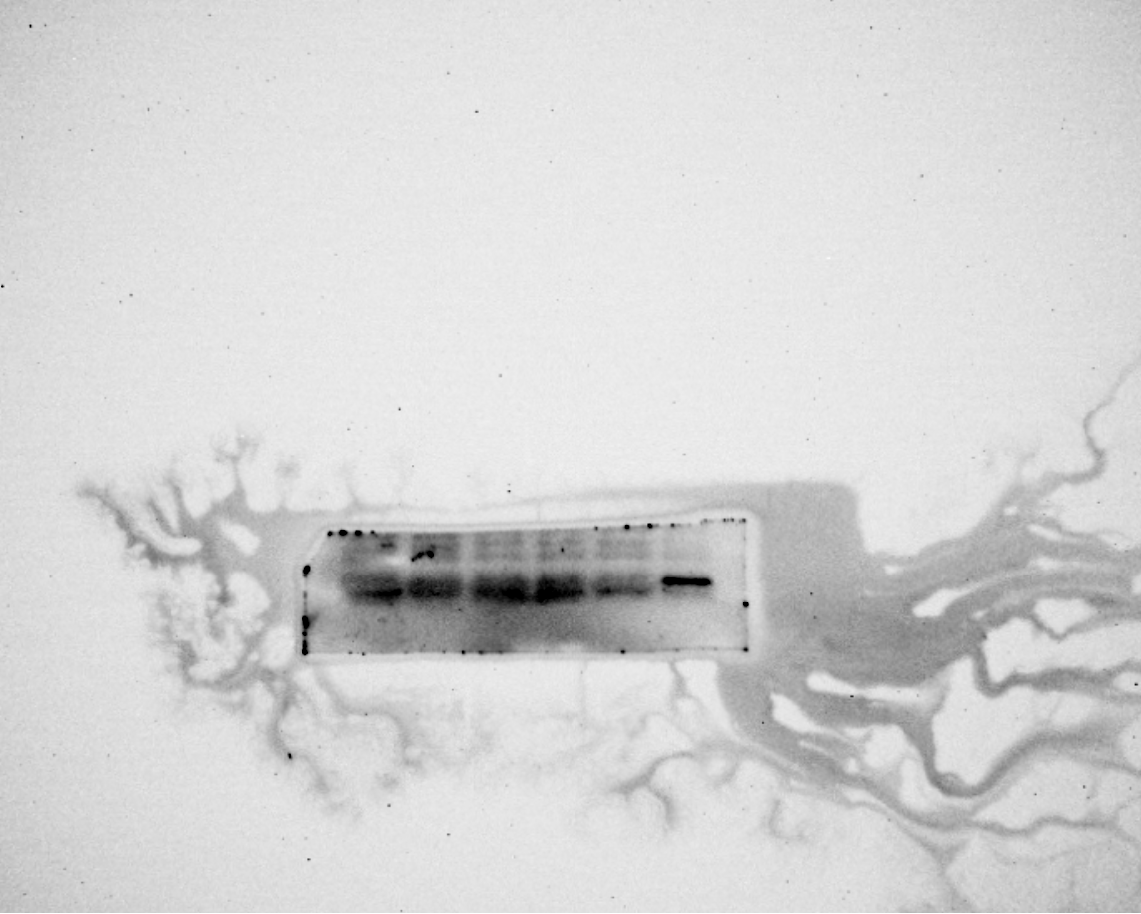

Supplement: Supplemental Information 3 — Cropped original blots in Figs. 6A–6C are shown in folders named REG4, SPINK1, LGALS1, respectively. Corresponding uncropped blots are shown in a PDF file named “uncropped blots”, where molecular weight ladders and target protein are clearly presented. Since small molecular weights of our target proteins (especially for SPINK1 ∼9kd), we did not get enough satisfying replicates in WB quantitative analysis. So relative mRNA levels were used to quantify expression of each gene in cell lines in Figs. 6A–6C (upper part), with WB results as a supplement reference (lower part). So only one WB result for each protein is provided. [file peerj-12-17350-s003.zip › REG4/jyt 2022-08-12 17h23m02s reg4(Chemiluminescence).tif]

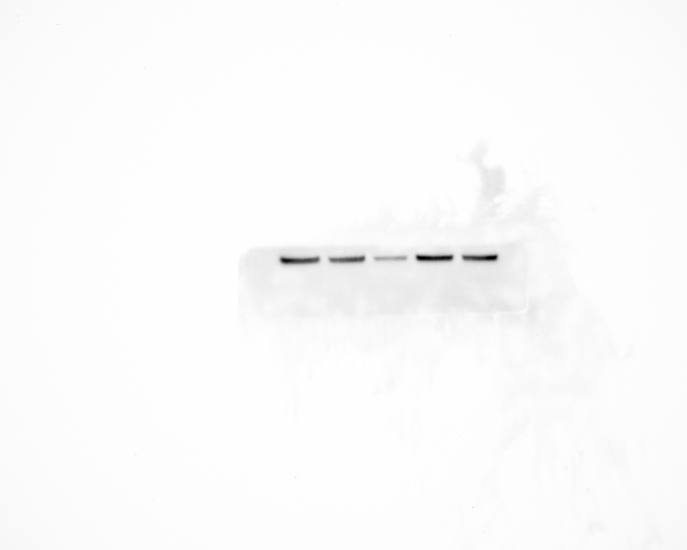

Supplement: Supplemental Information 3 — Cropped original blots in Figs. 6A–6C are shown in folders named REG4, SPINK1, LGALS1, respectively. Corresponding uncropped blots are shown in a PDF file named “uncropped blots”, where molecular weight ladders and target protein are clearly presented. Since small molecular weights of our target proteins (especially for SPINK1 ∼9kd), we did not get enough satisfying replicates in WB quantitative analysis. So relative mRNA levels were used to quantify expression of each gene in cell lines in Figs. 6A–6C (upper part), with WB results as a supplement reference (lower part). So only one WB result for each protein is provided. [file peerj-12-17350-s003.zip › SPINK1/jyt 2022-08-23 14h26m48s gapdh(Chemiluminescence).raw16.tif]

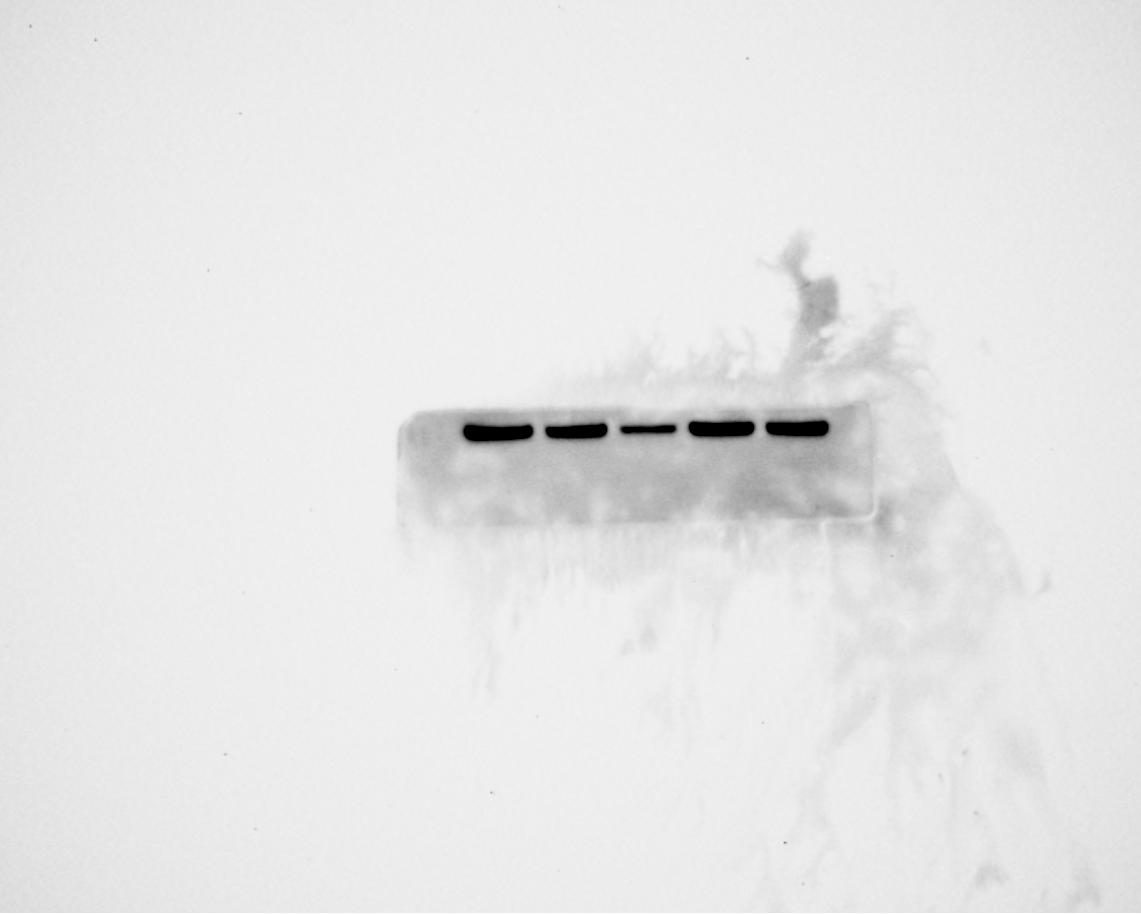

Supplement: Supplemental Information 3 — Cropped original blots in Figs. 6A–6C are shown in folders named REG4, SPINK1, LGALS1, respectively. Corresponding uncropped blots are shown in a PDF file named “uncropped blots”, where molecular weight ladders and target protein are clearly presented. Since small molecular weights of our target proteins (especially for SPINK1 ∼9kd), we did not get enough satisfying replicates in WB quantitative analysis. So relative mRNA levels were used to quantify expression of each gene in cell lines in Figs. 6A–6C (upper part), with WB results as a supplement reference (lower part). So only one WB result for each protein is provided. [file peerj-12-17350-s003.zip › SPINK1/jyt 2022-08-23 14h26m48s gapdh(Chemiluminescence).tif]

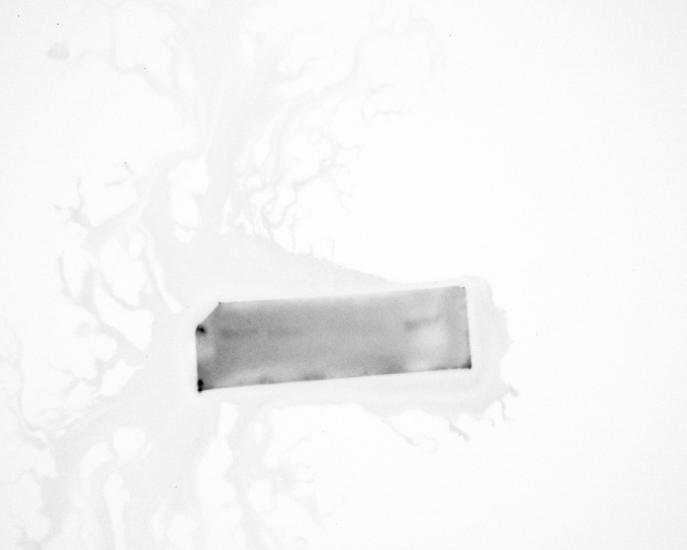

Supplement: Supplemental Information 3 — Cropped original blots in Figs. 6A–6C are shown in folders named REG4, SPINK1, LGALS1, respectively. Corresponding uncropped blots are shown in a PDF file named “uncropped blots”, where molecular weight ladders and target protein are clearly presented. Since small molecular weights of our target proteins (especially for SPINK1 ∼9kd), we did not get enough satisfying replicates in WB quantitative analysis. So relative mRNA levels were used to quantify expression of each gene in cell lines in Figs. 6A–6C (upper part), with WB results as a supplement reference (lower part). So only one WB result for each protein is provided. [file peerj-12-17350-s003.zip › SPINK1/jyt 2022-08-23 14h51m49s spink(Chemiluminescence).raw16.tif]

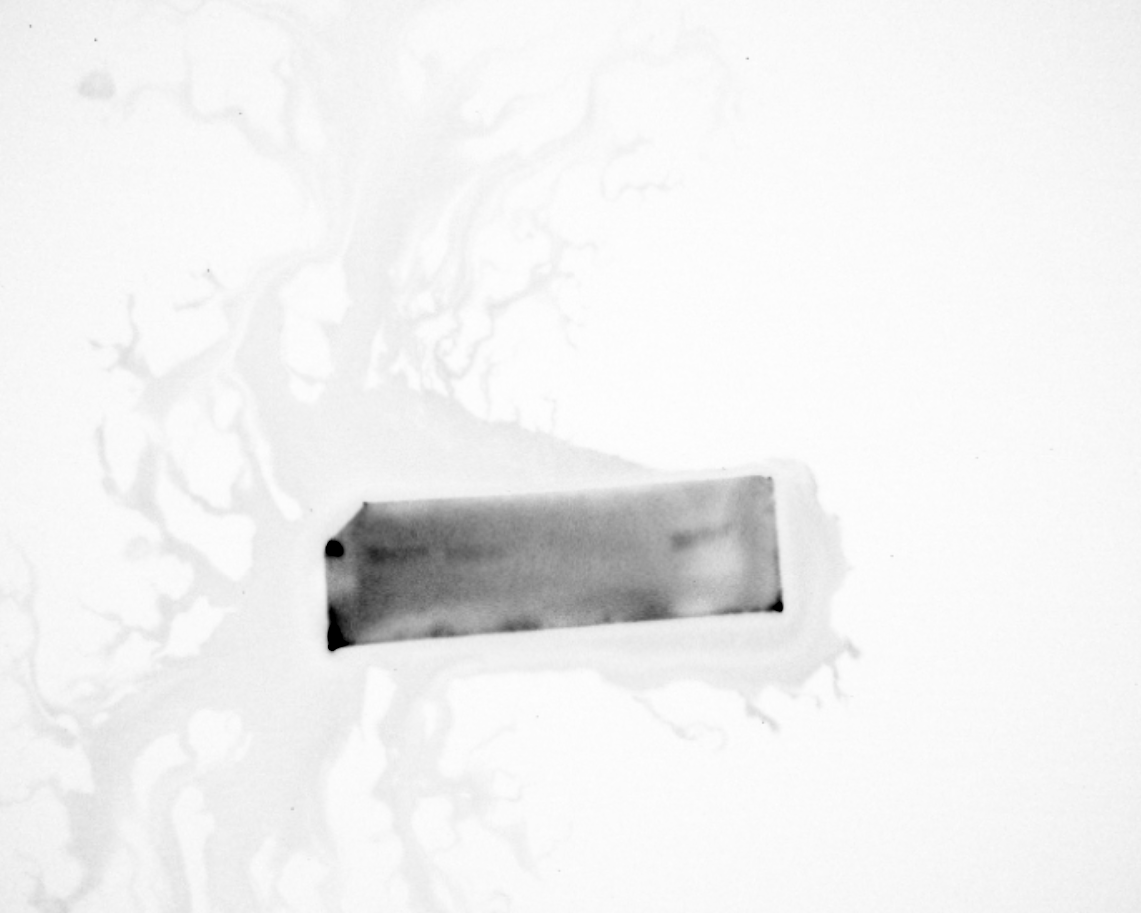

Supplement: Supplemental Information 3 — Cropped original blots in Figs. 6A–6C are shown in folders named REG4, SPINK1, LGALS1, respectively. Corresponding uncropped blots are shown in a PDF file named “uncropped blots”, where molecular weight ladders and target protein are clearly presented. Since small molecular weights of our target proteins (especially for SPINK1 ∼9kd), we did not get enough satisfying replicates in WB quantitative analysis. So relative mRNA levels were used to quantify expression of each gene in cell lines in Figs. 6A–6C (upper part), with WB results as a supplement reference (lower part). So only one WB result for each protein is provided. [file peerj-12-17350-s003.zip › SPINK1/jyt 2022-08-23 14h51m49s spink(Chemiluminescence).tif]
